# Genomic Analyses of *Weissella cibaria* W25, a Potential Bacteriocin-Producing Strain Isolated from Pasture in Campos das Vertentes, Minas Gerais, Brazil

**DOI:** 10.3390/microorganisms10020314

**Published:** 2022-01-28

**Authors:** Camila Gonçalves Teixeira, Rafaela da Silva Rodrigues, Ricardo Seiti Yamatogi, Anca Lucau-Danila, Djamel Drider, Luís Augusto Nero, Antônio Fernandes de Carvalho

**Affiliations:** 1 InovaLeite—Laboratório de Pesquisa em Leite e Derivados, Departamento de Tecnologia de Alimentos, Universidade Federal de Viçosa, Viçosa 36570 900, MG, Brazil; camila.g.goncalves.ufv@gmail.com (C.G.T.); rafaelasilva_mts@hotmail.com (R.d.S.R.); 2 Unité Mixte de Recherche (UMR) Transfrontalière BioEcoAgro1158, Univ. Lille, INRAE, Univ. Liège, UPJV, YNCREA, Univ. Artois, Univ. Littoral Côte D’Opale, ICV—Institut Charles Viollette, 59000 Lille, France; anca.lucau@univ-lille.fr (A.L.-D.); djamel.drider@univ-lille.fr (D.D.); 3 InsPOA—Laboratório de Inspeção de Produtos de Origem Animal, Departamento de Veterinária, Universidade Federal de Viçosa, Viçosa 36570 900, MG, Brazil; ryamatogi@ufv.br

**Keywords:** *Weissella*, genome sequencing, bacteriocins genes

## Abstract

*Weissella* is a genus containing Gram-positive, heterofermentative bacteria belonging to the lactic acid bacteria (LAB) group. These bacteria are endowed with promising technological and antimicrobial attributes. *Weissella cibaria* W25 was isolated from a dairy environment where raw milk cheeses are produced. Therefore, we sequenced and assembled the W25 draft genome sequence, which consists of 41 contigs totaling ~2.4 Mbp, with a G + C content of 45.04%. Then we carried out a comprehensive comparative genomic analysis with *W. cibaria* 110, known to produce the weissellicin 110 bacteriocin, and four other non-bacteriocin-producing *W. cibaria* strains.

## 1. Introduction

The study of microbial diversity in dairy and non-dairy environments plays a pivotal role in understanding the presence of these microorganisms in such ecosystems and their impact on the final product, especially when we refer to traditional and artisanal products. Each environment has unique and specific characteristics that favor and allow the development of different bacterial species [1].

Handmade cheeses and raw milk are considered potential sources of new strains of LAB [2]. The way these cheeses are made can determine the fermentation to be conducted by bacteria present in the grazing, animal skin, utensils, surfaces and other places that may come into contact with the cheese during production [3]. The study of the bacterial community present in artisanal cheeses revealed the presence of species that had not yet been related to cheeses and a high diversity of lactic bacteria with differentiated technological characteristics [4]. In addition, non-dairy environments such as grass, different types of silage and even animal skin have also been important sources of novel strains that have adapted and, therefore, can provide interesting features to be explored [5].

The diversity of *Weissella* isolated from dairy and non-dairy environments is of great interest for the enrichment in knowledge of this microorganism in the final products. The genus *Weissella* is composed of bacteria classified as Gram-positive, catalase-negative, non-spore forming, coccoid morphology or short bacilli. They belong to the group of LAB, mainly due to its production of lactic acid from the fermentation of carbohydrates [6]. The main purpose of this study is to announce and analyze the sequencing and annotation of the *Weissella cibaria* W25 genome and carry out a comprehensive comparative genomic analysis with *W. cibaria* 110, known to produce the weissellicin 110 bacteriocin, and four other non-bacteriocin-producing *W. cibaria* strains.

## 2. Materials and Methods

### 2.1. Bacterial Strain

The strain W25 was previously isolated from pasture sampled from a dairy farm located in the Campos da Vertentes region, in the southeast of the Minas Gerais state, Brazil. This strain was named “isolate id 25” by Teixeira et al. [7], identified as *Weissella cibaria* after sequencing of the gene 16S rRNA and characterized as possessing a technological potential due to its ability to coagulate milk and produce diacetyl and non-proteolytic. Of note, the strain presented antimicrobial activity against a panel of Gram-positive and Gram-negative foodborne pathogens [7].

### 2.2. Genome Sequencing and Assembly

The whole genome of *W. cibaria* W25 was sequenced with Nextera technology by a whole-genome shotgun strategy using the MiSeq v3 machine (Illumina, San Diego, CA, USA) by Neoprospecta (Florianópolis, SC, Brazil). The trimming was performed using the Trimmomatic v.0.36 [8] and the Phred value > 20. The raw read files were trimmed of adapter sequences and low-quality bases. After trimming, sequence reads were checked for quality using the fastQC v.0.11.5 [9] and then used for de novo genome assembly. Genome assembly was conducted by using MIRA Assembler v.4.9.6 [10], mode “genome, accurate”. The assembling quality was determined with QUAST v.5.0.2 [11], and ContEst16S was used to check contamination [12].

### 2.3. Genome Annotation and Analysis

Gene prediction and annotation were performed using the Rapid Prokaryotic Genome Annotation (PROKKA) v.1.14.5 [13], executed with default parameters and also performed by RAST automated web server [14]. To identify secondary metabolite biosynthetic gene clusters and bacteriocins, we used the antiSMASH v.6.0 [15] and the BAGEL4 webserver [16]. Moreover, the research for plasmid was evaluated by plasmidFinder [17] and the web tool PathogenFinder [18] was used to check the presence of potential virulence factors.

### 2.4. Phylogenetic Analyses

The identification of the genus and species was carried out using KmerFinder [19,20] and the Type (Strains) Genome Server (TYGS) [21]. The phylogenetic trees were visualized and edited using the online tool iTol v.6 [22].

### 2.5. Comparative Genomic

To establish the relationship between *W. cibaria* W25 and other members of this species in the bacteriocin production context we selected four published non-bacteriocins-producing *W. cibaria* strains and *W. cibaria* 110 known to produce the weissellicin 110 bacteriocin (Table 1). The genomes obtained from the GenBank were annotated using Prokka before being subjected to analyses in order to standardize the annotations.

The draft genome was submitted to the Type (Strains) Genome Server (TYGS) to confirm the genus and species. Moreover, to establish the genetic similarity between all strains, analysis was done with Digital DDH (DNA–DNA hybridization) similarities based on the GGDC (Genome-to-Genome Distance Calculator) web server, version 3.0 [23]. The core genome of each group was determined with OrthoVenn2 (e-value of 10^−5^) [24], and the CGView Server [25] was used for comparative genome analysis using BLAST with default parameters.

### 2.6. Availability of Nucleotide Sequence Data

This Whole Genome Shotgun project was deposited at DDBJ/ENA/GenBank under the accession JAFNKE000000000. The version described in this paper is version JAFNKE010000000. The raw sequencing data were submitted to the Sequence Read Archive (SRA) database under accession number SRR16076638.

## 3. Results and Discussion

### 3.1. Genome Sequencing, Annotation and Analysis

The genome features comparison between *W. cibaria* W25, *W. cibaria* 110, *W. cibaria* B3b, *W. cibaria* ffPR, *W. cibaria* JCM 12,495 and *W. cibaria* MG1 and the predicted genes are presented in Table 1. The whole-genome sequencing of *W. cibaria* W25 resulted in maximum size of reads for the forward sequence of 305 and for the reverse sequence of 205 and with a total number of sequences of 2,906,916 bp. After genome assembly using the MIRA software, we obtained a draft genome with 41 contigs, N_50_ 202,649 bp and maximum length of 331,445 bp (contigs over 500 bases).

The genome of *W. cibaria* W25 contains 2,412,435 bp, which is very similar to the genome of *W. cibaria* MG1, and slightly bigger than *W. cibaria* 110, *W. cibaria* ff3PR and *W. cibaria* JCM 12495. However, the GC contents are very similar between all of them, varying from 44.7 to 45.1%. Genome annotation using Prokka identified a total of 2190 of coding DNA sequences (CDS) in the genome of *W. cibaria* W25. The amount of CDS were more abundant in our genome strains than in the genome of *W. cibaria* JCM 12,495 and less abundant than in the other genomes in this study. *W. cibaria* W25 presented the highest quantity of tRNA and rRNA with 11 copies of 5S ribosomal RNA (rRNA) genes, 3 copies of 16S and 1 single copy of 23S rRNA genes.

It is worth mentioning that draft genome assemblies often include an incorrect number of rRNA genes due to assembly artifacts.

No plasmid gene was detected by plasmidFinder in *W. cibaria* W25, and this input organism was predicted as a non-human pathogen by PathogenFinder. These results are an indication for the safe use of this strain for future human consumption as a probiotic or as a bioprotective culture in food, for example.

According to AntiSMASH, *W. cibaria* W25 possesses two putative bacteriocin gene clusters, one lassopeptide (MicJ25) and one RiPP-like bacteriocin_IIc. Of note, the Bagel4 software did not allow identification of any bacteriocin gene. Previously, we showed that *W. cibaria* W25 has a narrow spectrum of inhibition against the most common foodborne pathogens [7], reinforcing the idea that this strain is a suitable probiotic candidate. Li et al. (2017) [26] showed that *W. cibaria* 110 presents similar results showing a large spectrum of inhibition against other LAB, but the bacteriocin weissellicin 110, produced by *W. cibaria* 110, unlike most class II bacteriocins, has no inhibitory activity against *Listeria monocytogenes*.

### 3.2. Phylogenetic Analyses and Comparative Genomic

**Figure 1:**
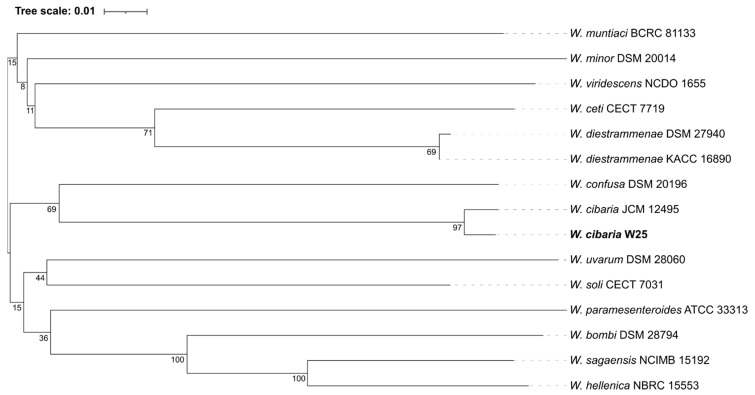
Tree inferred with FastME 2.1.6.1 [29] from GBDP distances calculated from genome sequences. The branch lengths are scaled in terms of GBDP distance formula d_5_. The numbers above branches are GBDP pseudo-bootstrap support values >60% from 100 replications, with an average branch support of 55.9%. The tree was rooted at the midpoint [30].

Whole-genome-based phylogeny of the *W. cibaria* W25 was constructed using several genome sequences from *Weissella* species-type strains, including complete and draft genomes (Figure 1), by the TYGS web server. Figure 1 shows the formation of two major clusters, cluster one comprised six *Weissella* strains and cluster two all the other strains including the one in study. In addition, Figure 1 shows that *W. cibaria* W25 and *W. cibaria* JCM 12,495 are phylogenetically closely related. This result, along with the one from KmerFinder software, confirmed the genus *Weissella* and the species *cibaria* for the strain W25, as previously announced [7].

The digital DDH genomic similarity (Table 2) revealed that between the strains tested in this study *W. cibaria* W25 possesses more similarity with *W. cibaria* JCM 12,495 (87.10%), a non-bacteriocin producer, and less similarity with *W. cibaria* 110 (63.80%), a strain known as a bacteriocin producer.

**Figure 2:**
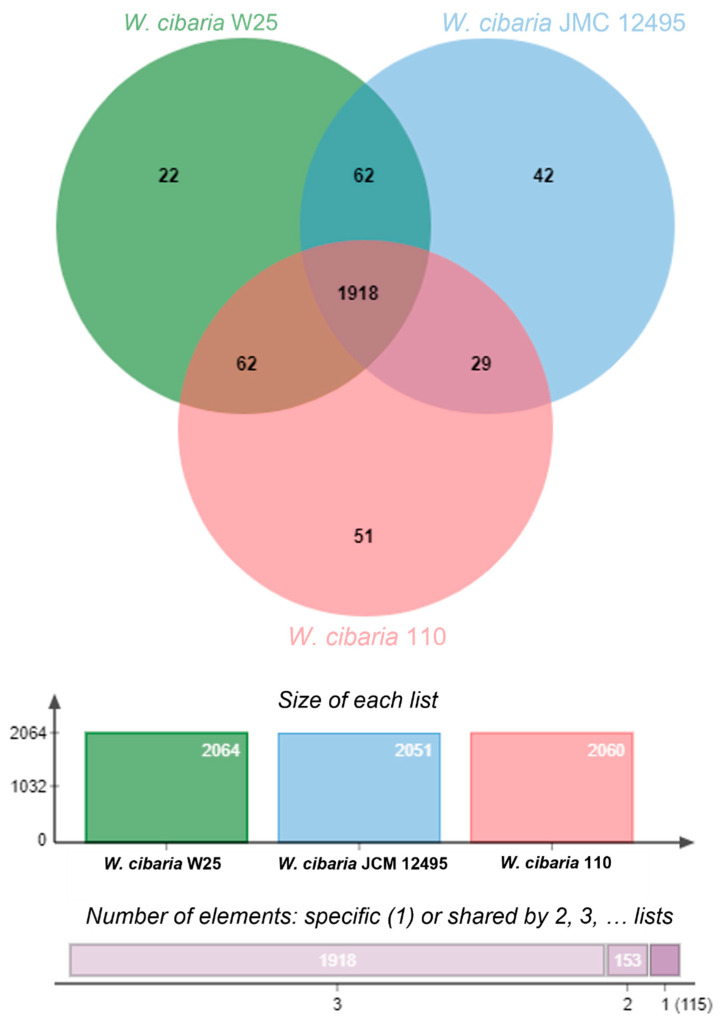
Venn diagram showing the protein coding genes or pseudogenes in each sequenced of *W. cibaria* W25, *W. cibaria* 110 and *W. cibaria* JMC 12495. Overlapped regions represent shared proteins and the numbers in the non-overlapped regions indicate the unique protein coding genes or pseudogenes.

**Figure 3:**
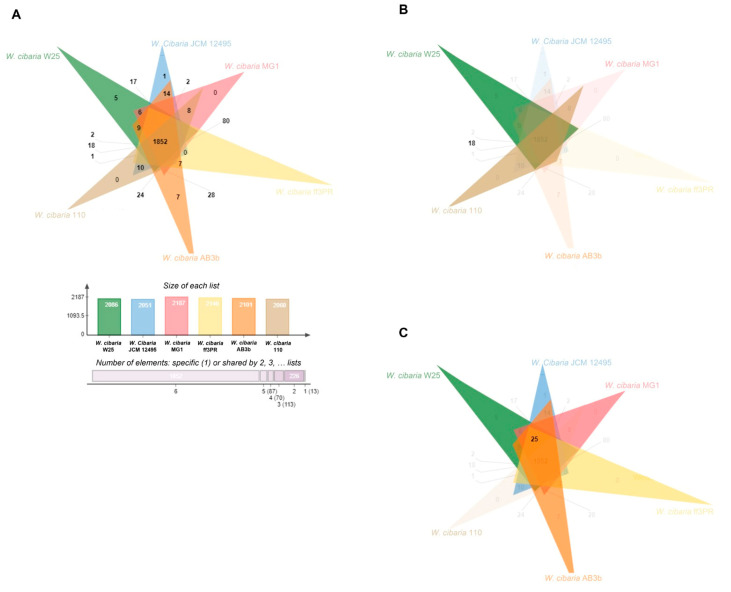
Venn diagrams showing the protein coding genes or pseudogenes of six *W. cibaria* strains. Overlapped regions represent shared proteins and the numbers in the non-overlapped regions indicate the unique protein coding genes or pseudogenes. (**A**) normal Venn diagram; (**B**) Venn diagram highlighting the cluster between *W. cibaria* W25 and *W. cibaria* 110; (**C**) Venn diagram highlighting the clusters between *W. cibaria* W25 and the non-bacteriocin-producing strains.

According to the Venn diagram from OrthoVenn2, *W. cibaria* W25 shares the same quantity of protein cluster genes with *W. cibaria* 110 and *W. cibaria* JCM 12,495 (Figure 2) when comparing just the three of them. When we compared with all the strains used in this study, *W. cibaria* W25 shares more unique protein cluster genes [18] with *W. cibaria* 110 (Figure 3B) than with the others. Besides that, *W. cibaria* W25 shares 25 with all the non-bacteriocin-producing strains (Figure 3C). There were 1852 protein cluster genes conserved in all *W. cibaria* strains, and five of them are unique to *W. cibaria* W25 (Figure 3A) with 10 paralogs. Among the five protein clusters coding genes, the OrthoVenn2 identified three of them, one related to the lipopolysaccharide biosynthetic process, one to the O antigen biosynthetic process and one to oxidoreductase activity. Li et al. (2017) [25] also compared *W. cibaria* 110 with four other strains (*W. cibaria* MG1, *W. cibaria* AB3b, *W. cibaria* ff3PR and *W. cibaria* KACC11862) and the comparative genomic analysis also showed the presence of unique genes that encoded the novel bacteriocin weissellicin 110 and defense system.

**Figure 4:**
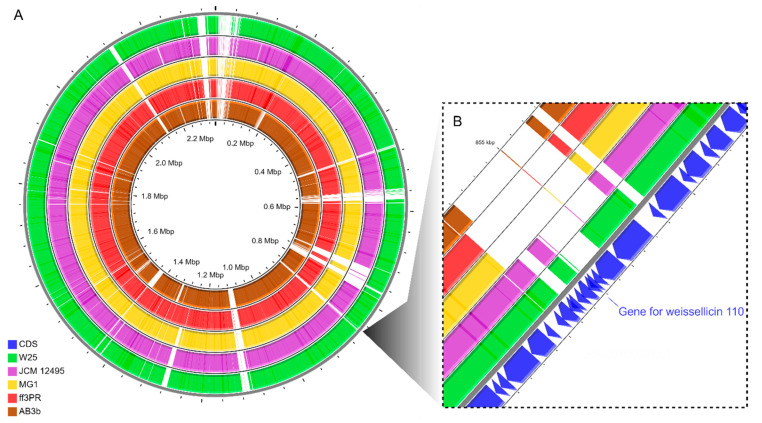
Comparative genome map generated using CGView server, showing a full circular map obtained using BLAST (**A**) and an expanded view of the weissellicin 110 region (**B**). The rings in (**A**) and (**B**) in green, purple, yellow, red and brown indicate the *W. cibaria* strains W25, JCM 12495, MG1, ff3PR and AB3b, respectively, that were compared with the *W. cibaria* 110. The blue ring in (**B**) indicates the coding sequences (CDS) of the genome from *W. cibaria* 110.

We constructed a comparative genetic map using the CGview server to help demonstrate the similarity between the strains (Figure 4). We used *W. cibaria* 110 as the reference for comparison because it is a well-known bacteriocin-producing strain. Moreover, gene confirmation for weissellicin 110 was performed by the BLASTn program at the National Center for Biotechnology Information (NCBI) using the deposited sequence under accession number LC010242 and the predicted genes of the genome from *W. cibaria* 110. We obtained results to query coverage and percent identity of 100%. Among the regions that showed to be similar between *W. cibaria* W25 and *W. cibaria* 110, we observed that the strain under study presented a similar and more complete region when compared to the region containing weissellicin 110 than the non-bacteriocin-producing strains (Figure 4B). This indicates that among the shared genes between *W. cibaria* W25 and *W. cibaria* 110 there is one related to the production of bacteriocin but they are not completely the same, which indicates that perhaps in this region there are genes for a bacteriocin, however, they are distinct from weissellicin 110.

## 4. Conclusions

According to the bioinformatics results obtained in this study, *W. cibaria* W25 has great potential to be used for human consumption since it was predicted as a non-human pathogen. In addition, despite *W. cibaria* W25 showing more genomic similarity with *W. cibaria* JCM 12,495 (according to DDH similarly), OrthoVenn2 showed that it has its unique protein cluster genes which can be related with the bacteriocin genes indicated by AntiSMASH, confirming the possibility of producing two different bacteriocins.

## Figures and Tables

**Table 1:** Comparison of the genomic feature of *W. cibaria* W25 with a bacteriocin-producing *W. cibaria*, strains 110, and four other non-bacteriocin-producing strains.

Genome Feature	W25	110	AB3b	ff3PR	JCM 12495^T^	MG1
Accession ^a^	JAFNKE000000000	LRRC00000000	JWHT00000000	JWHV00000000	BJEF00000000	JWHU00000000
Reference	This study	[26]	[27]	[27]	[28]	[27]
Contigs	41	18	88	60	25	44
Size (pb)	2,412,435	2,347,049	2,465,158	2,357,128	2,323,953	2,436,232
GC content (%) ^b^	45.04	44.9	44.7	44.9	45.1	44.7
CDS	2190	2209	2348	2228	2124	2284
tRNA	84	76	67	70	77	62
rRNA	15	5	7	5	9	4
tmRNA	1	1	1	1	1	1
Repeat region	1	1	0	0	0	0

^a^ GenBank accession number; ^b^ using RAST program.

**Table 2:** Genetic similarity between *W. cibaria* W25 and other strains using Digital DDH (DNA–DNA hybridization) similarities based on the GGDC (Genome-to-Genome Distance Calculator). The recommended formula 2 was chosen for the inferred distances.

Strains	DDH	Prob. DDH ≥ 70%	G + C Difference
*W. cibaria* 110	63.8	63.95	0.18
*W. cibaria* AB3b	72.6	82.63	0.36
*W. cibaria* ff3PR	71.7	81.21	0.17
*W. cibaria* JCM12495	87.1	94.69	0.11
*W. cibaria* MG1	71.6	81.02	0.29

## Data Availability

Not applicable.
